# An overview of the performance of AI in fracture detection in lumbar and thoracic spine radiographs on a per vertebra basis

**DOI:** 10.1007/s00256-024-04626-2

**Published:** 2024-02-27

**Authors:** Oppenheimer J., Lüken S., Geveshausen S., Hamm B., Niehues S. M.

**Affiliations:** Charité Universitätsmedizin Berlin, Klinik für Radiologie, Campus Benjamin FranklinHindenburgdamm 30, 12203 Berlin, Germany

**Keywords:** Artificial intelligence, Radiography, Trauma, Computer-aided diagnosis

## Abstract

**Purpose:**

Subtle spinal compression fractures can easily be missed. AI may help in interpreting these images. We propose to test the performance of an FDA-approved algorithm for fracture detection in radiographs on a per vertebra basis, assessing performance based on grade of compression, presence of foreign material, severity of degenerative changes, and acuity of the fracture.

**Methods:**

Thoracic and lumbar spine radiographs with inquiries for fracture were retrospectively collected and analyzed by the AI. The presence or absence of fracture was defined by the written report or cross-sectional imaging where available. Fractures were classified semi-quantitatively by the Genant classification, by acuity, by the presence of foreign material, and overall degree of degenerative change of the spine. The results of the AI were compared to the gold standard.

**Results:**

A total of 512 exams were included, depicting 4114 vertebra with 495 fractures. Overall sensitivity was 63.2% for the lumbar spine, significantly higher than the thoracic spine with 50.6%. Specificity was 96.7 and 98.3% respectively. Sensitivity increased with fracture grade, without a significant difference between grade 2 and 3 compression fractures (lumbar spine: grade 1, 52.5%; grade 2, 72.3%; grade 3, 75.8%; thoracic spine: grade 1, 42.4%; grade 2, 60.0%; grade 3, 60.0%). The presence of foreign material and a high degree of degenerative changes reduced sensitivity.

**Conclusion:**

Overall performance of the AI on a per vertebra basis was degraded in clinically relevant scenarios such as for low-grade compression fractures.

## Purpose

Spinal compression fractures are one of the most common fracture types, especially in the elderly population [1, 2]. With radiographs being the initial imaging method of choice, radiologists are presented with spine radiographs with inquiry for fracture on a daily basis. Interpretation of these radiographs may be difficult, as many radiographs show severe degenerative changes, patient mobility and therefore image quality may be compromised, or prior orthopedic surgery may have taken place. Missing fractures in these radiographs may result in delays in treatment, possibly resulting in further complications [3]. Slight compression fractures in particular may easily be missed [4–7].

An increasing number of AI tools are available for diagnostic assistance in radiology. One such tool is Gleamer BoneView®, which aids in fracture detection on X-rays. The tool has shown overall good sensitivity and specificity for fractures, however, vertebral fractures lagged compared to other anatomic regions in a study by Guermazi et al. While overall sensitivity and specificity for all regions was 88%, this was reported to drop to 77% specificity and 80% sensitivity in the thoracolumbar spine [8]. Similar results were shown in a study by Oppenheimer et al., with sensitivity at 89% and specificity at 62% [9]. These studies measured the AI performance only on a per case basis, not analyzing each potential separate vertebral fracture in the radiograph. Additionally, it was also not measured which influence the grade of compression had on sensitivity and specificity.

The Genant classification allows for a semi-quantitative classification of vertebral compression fractures by percentage of height loss as well as fracture type. Hereby grades 1-3 (mild, moderate, severe) are distinguished by the percentage of height loss. Grade 1 fractures show a height loss of less than 25%, grade 2 fractures 25–50%, and grade 3 fractures greater than 50%. Fracture type is classified into anterior (wedge type), middle (crush type), and posterior compression fractures [10].

We aim to retrospectively test the sensitivity and specificity of a commercially AI system for fracture detection (Gleamer BoneView®) for vertebral compression fractures in relation to the Genant classification system on a per vertebrae basis as a primary result. A high sensitivity and specificity for subtle fractures (Genant 1) is necessary for the software to aid the radiologist. Additionally, we aim to test the performance of the AI on multiple subsets of data that are regularly encountered in everyday practice, but may impair the diagnosis. Therefore, we will test the performance when comparing patients where surgery or cement kyphoplasty was previously performed and in patients with various stages degenerative changes of the spine. We will also compare the performance for acute versus non-acute compression fractures.

## Materials and methods

### Data collection

**Image 1:**
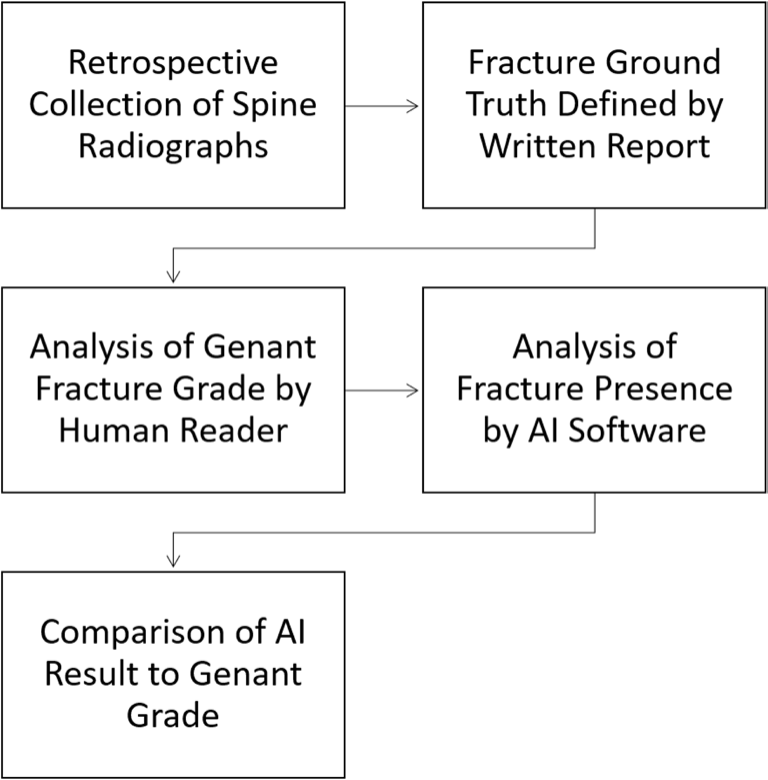
General overview of the study design

An overview of the study design is shown in Image 1. Lumbar and thoracic spine radiographs with inquiry for fracture were retrospectively collected from a level 3 trauma center’s PACS-Database from February 2022 to June 2022. The search was performed in our clinic’s RIS-System (GE Centricity RIS-I 7.0, GE Healthcare, Chicago, IL, USA), filtering for the exam types “lumbar spine radiograph” and “thoracic spine radiograph.” Only radiographs with inquiry for fracture were included, spine radiographs acquired for other inquiries such as degenerative changes or pre- and post-surgery imaging were excluded. Cervical spine radiographs were excluded, as these are not supported by the software for analysis. Exams not including a sagittal image were excluded. A thoracic and lumbar spine exam may have been acquired for the same patient in some cases, if so, each was included as a separate entity (Table 1).

**Table 1:** Inclusion and exclusion criteria

***Inclusion criteria***	***Exclusion criteria***
Thoracic or lumbar spine radiograph	Cervical spine radiograph
At least sagittal image acquired	No sagittal image
Inquiry for new fracture	Other inquiries

**Image 2:**
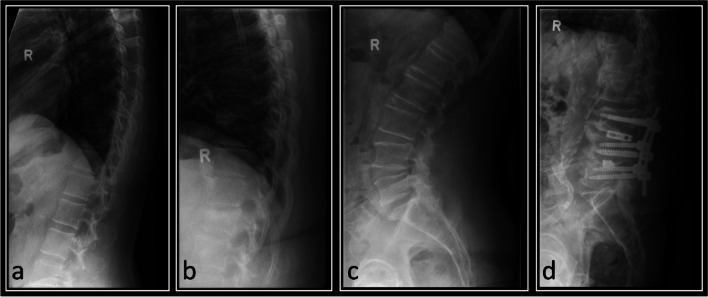
**a**–**d** From left to right: lateral radiographs of the thoracic (**a**, **b**) and lumbar spine (**c**, **d**) from different patients. Image **a** showing no mentionable degenerative changes, **b** showing mild changes, **c** moderate, and **d** severe changes

Patient age and gender were noted. The clinical indication for the imaging was broadly categorized into seven categories, (1) pain without known cause, (2) falls, (3) assault, (4) other trauma, (5) osteoporosis, (6) metastatic bone disease, and (7) other. Each patient’s exam was subjectively semi-quantitatively categorized by degree of degenerative change, on a scale from 0 (none) to 3 (severe) (see Image 2 a–d). Mild degenerative changes included minimal height loss of the intervertebral space and minimal sclerosis of the vertebral end plates. Moderate changes included height loss of the intervertebral space over 50% in one or more segments, extensive end plate sclerosis and/or non-bridging osteophytes or syndesmophytes of some vertebrae. Severe changes included complete loss of the intervertebral space and/or bridging osteophytes or non-bridging osteophytes of most vertebrae.

Presence or absence of fracture was defined by the written report as a consensus of two radiologists, at least one of which was board-certified radiologist with over 5 years of experience in musculoskeletal radiology. Where available, cross-sectional imaging was used as the gold standard. Each fracture was graded on the Genant-Scale by measured relative height loss and fracture type, in the sagittal radiograph, as described above. Where cross-sectional imaging was available, and height loss and fracture type were classified by these images. It was noted if any foreign orthopedic material was present in the vertebra, and this material was categorized into (1) only metal such as screws, (2) only cement, (3) a combination of screws and cement, or (4) full vertebral replacement. Each fracture was classified as acute or non-acute by imaging characteristics, clinical information, and where available comparison to prior imaging. Typical imaging characteristics for acute fractures include increased density of the endplate due to trabecular impaction and endplate disruption while chronic fractures. Non-acute fractures typically show callus formation along the endplate. However, these characteristics have an overall low specificity when compared to the gold standard of MRI [11, 12]. To better classify the acuity, available clinical information, i.e., acute new pain or recent trauma was included where available. We realize that without MRI imaging for all patients the results of this categorization should be regarded with caution.

### AI analysis

**Image 3:**
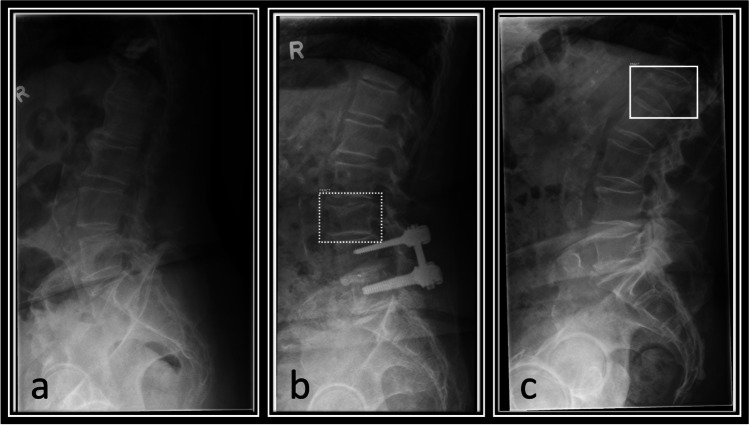
**A**–**c** From left to right with markings by the AI software: lateral radiographs of the lumbar spine from different patients. Overlay of images returned by the AI showing no regions of interest (**a**), a dashed-line bounding box (**b**) where a fracture is deemed possible and a through line bounding box (**c**) where a fracture is deemed likely. **b** and **c** show true positive results by the AI

Each included exam was then sent to our on-site server for analysis by the fracture detection AI (BoneView© Version 1.2.0, Gleamer, Paris, France). Results were returned within minutes as additional images in the clinic’s PACS System (Phönix PACS MERLIN Diagnostic Workcenter Version 7.0, Phönix-PACS GmbH, Freiburg, Germany). One image shows the overall result for the exam as either “Positive,” “Doubt,” or “Negative” as well as an image overlay for each original image included in the exam marking the fractures. These show either images with no results (“Negative,” Image 3a), with through line bounding boxes marking fractures the AI deems as likely having a fracture (“Positive,” Image 3c) or dashed-line bounding boxes, where the AI deems a fracture possible (“Doubt,” Image 3b). The AI has a threshold of “Doubt” at 50–89% confidence and “Positive” at 90% and above [8]. For spine radiographs, these bounding boxes are generally placed around an entire vertebra. For each marked vertebra it is noted if it is marked as “Positive” or “Doubt,” or if the vertebra is unmarked by the software in the a-p and sagittal images. Positive and doubt results were classified as fracture positive by the AI for further analysis. In rare cases where the bounding box is between vertebra, this is not noted as any marking on the vertebra (see Image 3).

### Statistical analysis

Results of the AI software were compared on a per vertebrae basis to the defined Genant classification. Sensitivity, specificity, positive, and negative predictive value were calculated for the overall dataset and then for each of the nine classifications defined by Genant (wedge-type grade 1–3, crush type grade 1–3, posterior compression grade 1–3). Results are primarily reported only in comparison to the side view radiograph, as this view defined the Genant category. Further analysis was provided for the entire set with the results of the ap-images included, only including vertebra depicted on both views.

Sub-group analysis was performed for old vs. new fractures, for grade of degenerative change, as well as for vertebra including or not including foreign orthopedic material. Results were compared by gender as well as for age cohorts in intervals of 20 years.

All exams and vertebrae were regarded as separate for purposes of this analysis. Lumbar and thoracic spine results were calculated separately. All results are shown with the range for 95% confidence intervals (±). Data analysis and documentation were performed with Excel 365 (Microsoft Corporation, Redmond, WA; USA) and IBM SPSS Statistics 29 (IBM, Armonk, NY, USA). Means were compared by independent t-tests or ANOVA where applicable. Significance was defined as *p* < 0.05.

## Results

### Patient cohort

A total of 512 exams from 400 patients (357 lumbar spine and 155 thoracic spine) were included. Average patient age was 67.5 years (± 1.6 years; range 19–100). Two patients were in the age cohort 0–19 years, 62 in the cohort 20–39 years, 88 in the group 40–59 years, 181 in the group 60–79 years, 178 in the cohort of 80–99 years, and one patient was in the group of 100 years of age and older. 37.4% of patients were male. The average age of female patients was 70.1 years (range 19–100), for male patients 63.2 years (range 21–93). 73.7% of exams were acquired in standing position. Cross-sectional imaging was available for 107 (20.9%) cases. Three hundred four exams were ordered for pain without trauma, 155 for falls, 6 for assaults, 13 for other trauma, five for follow-up in patients with known osteoporosis, and 26 for known metastatic bone disease. Two exam orders were classified as other. Sixty-eight exams showed spinal imaging without noteworthy degenerative changes, 142 with initial degenerative changes, 149 with moderate, and 152 with severe degenerative changes. One hundred three patients had radiographic signs of osteoporosis; 163 patients had diagnosed osteoporosis.

Five lumbar spine and eight thoracic spine radiographs had to be excluded, as they were rejected by the AI software for analysis as being of unsupported anatomical regions (either being classified by the AI as chest or abdominal exams).

A total of 2504 vertebrae were included in the lumbar spine exams (with the sixth thoracic to the fifth lumbar vertebra being included in images). Of these, 2181 had no fracture. A total of 1610 vertebrae in the thoracic spine radiographs were included (with imaging of the first thoracic to the fourth lumbar vertebra being included in images), of which 1438 had no fracture. The distribution of fractures by Genant classification is shown in Table 2. No radiographs included posterior fractures, as these often present with concordant neurologic deficits, cross-sectional imaging is the initial modality of choice where these are suspected.

**Table 2:** Fracture distribution by Genant classification and by thoracic and lumbar spine radiographs

	Lumbar	Thoracic	Total
No fracture	2181	1438	3619
Grade 1 Wedge	88	45	133
Grade 2 Wedge	74	28	102
Grade 3 Wedge	54	31	85
Grade 1 Crush	72	40	112
Grade 2 Crush	27	17	44
Grade 3 Crush	8	11	19
Total	2504	1610	4114

### Sensitivity and specificity

Overall sensitivity of the AI on lateral images was 63.2% in lumbar spine radiographs, significantly better than thoracic spine images at 50.6% sensitivity (*p* = 0.01). Specificity was 96.7% and 98.3% respectively. The sensitivity of the AI improved somewhat with a higher fracture grade; however, there was no significant difference between grade 2 and 3 fractures (lumbar spine: *p* = 0.90; thoracic spine: *p* = 0.99). Difference between grade 1 to 2 and 3 was significant in the lumbar spine only (lumbar spine: *p* (1,2) = 0.01 and *p* (1,3) = 0.01; thoracic spine: *p* (1,2) = 0.13, *p* (1,3) = 0.62). The AI showed slightly, non-significant, better results for wedge fractures in lumbar spine radiographs versus crush fractures (64.4 vs. 60.8%; *p* = 0.53), with less difference between the two groups in thoracic radiographs (51.9 and 50.0%; *p* = 0.81). Sensitivity was lowest for grade 1 crush fracture in the thoracic spine (40.0%) and highest for grade 2 crush fractures in the lumbar spine (81.5%). Sensitivities for each fracture grade are shown in Table 3. With the addition of ap-images sensitivity significantly increased for lumbar spine radiographs to 72.4% (± 4.9; *p* = 0.01). Improvement was shown in thoracic spine radiographs also, albeit non-significant, with sensitivity increasing to 60.6% (95% CI ± 7.5; *p* = 0.08). Specificity remained high at 94.2% (± 1.0) and 94.0% (± 1.3) respectively.

**Table 3:** Overall sensitivity and specificity and sensitivities by fracture grade for lumbar and thoracic spine (in percent; ± 95% confidence interval)

	Lumbar spine (in %)	Thoracic spine (in %)
Sensitivity lateral only	**63.2** (± 5.3)	**51.2** (± 7.5)
Specificity lateral only	**96.7** (± 0.8)	**98.3** (± 0.7)
Sensitivity lateral + ap	**72.4** (± 4.9)	**60.6** (± 7.5)
Specificity lateral + ap	**94.2** (± 1.0)	**94.0** (± 1.3)
*Sensitivity for:*		
Grade 1	**52.5** (± 7.7)	**42.4** (± 10.5)
Grade 2	**72.3** (± 8.7)	**60.0** (± 14.3)
Grade 3	**75.8** (± 10.7)	**60.0** (± 14.8)
Wedge	**64.4** (± 6.4)	**51.9** (± 9.6)
Crush	**60.8** (± 9.3)	**50.0** (± 11.9)
Grade 1 Wedge	**53.4** (± 10.4)	**46.7** (± 14.6)
Grade 2 Wedge	**68.9** (± 10.6)	**57.1** (± 18.3)
Grade 3 Wedge	**75.9** (± 11.4)	**58.0** (± 17.4)
Grade 1 Crush	**51.4** (± 11.5)	**40.0** (± 15.8)
Grade 2 Crush	**81.8** (± 14.7)	**64.7** (± 22.7)
Grade 3 Crush	**75.0** (± 30.0)	**60.0** (± 13.1)

### Age and gender

Lumbar spine images included 103 fractures in male patients resulting in a sensitivity of 62.1% and specificity of 96.0%. In female patients, 220 fractures were included, with a sensitivity of 63.6% and specificity of 97.1%. For thoracic spine radiographs, the gender difference was more pronounced, with a sensitivity of 29.4% in males and 56.5% for females, however there were more than twice as many analyzed vertebrae in the female cohort. Sensitivity was 97.7% and 98.5% respectively.

The age group of 0–19 and 100 and above were not separately analyzed, as they were included too few patients. For lumbar spine images, sensitivity varied between 50.0% in the age group 20–39 to 64.9% in the 80–99 group. Specificity was above 95% in all groups. The age group of 20–39-year-olds only included a total of 12 fractures, eight of which were grade 1 wedge fractures. The difference was even larger in the thoracic spine radiographs, where the age group 20–39 had a sensitivity of only 25%, however with only 4 fractures total. Sensitivity was highest for the 60- to 79-year-old group with 54.7%. Specificity also remained above 95% in all groups.

### Fracture acuity

Of the lumbar spine fractures, 244 (75.5%) were classified as non-acute by either imaging characteristics or availability of prior studies. Seventy-four were classified as acute fractures (22.9%), five fractures were not classifiable. Sensitivity was similar for both groups; for acute fractures at 62.2% (± 11.1) and for non-acute fractures at 63.1% (± 6.1).

For thoracic spine fractures, 150 were classified as non-acute (87.2%) and 14 (8.1%) as acute, and 18 were not classifiable. Sensitivity for non-acute fractures (53.3% ± 8.0) was better than for acute fractures (42.9% ± 25.9).

### Foreign material

One hundred twenty-three (4.9%) lumbar spine vertebrae had foreign material present after orthopedic surgery, of which 59 were metal screws, 45 were after treatment with cement kyphoplasty, 32 were a combination of screws and cement, and 7 were complete vertebral replacements. With foreign material present, sensitivity dropped to 58.8% (± 13.5) and specificity to 89.1% (± 6.4). Sensitivity was 64.2% (± 5.9) and specificity 96.6% (± 0.1) when no material was present in the vertebra.

In the thoracic spine radiographs, 80 vertebra (5.0%) had foreign material present, half of which with screws only, 25 with cement, 8 with a combination, and 7 vertebral replacements. With material present, sensitivity was 40.5% (± 15.8) and specificity 93.0% (± 7.6). Without material, sensitivity increased to 53.73% (± 8.4) and specificity to 98.4% (± 0.7).

### Degenerative changes

Sensitivity and specificity compared by degree of degenerative changes showed a wide range. Patients with no degenerative changes were underrepresented in the dataset, resulting in a wide confidence interval. Sensitivity was 50.0% (± 26.2) and 50.0% (± 69.3) for the lumbar and thoracic spine respectively. For lumbar spine radiographs, sensitivity otherwise decreased with increasing degenerative change, thoracic spine radiographs showed a higher sensitivity with moderate changes compared to mild changes. Full results are shown in Table 4.

**Table 4:** Sensitivities and specificities by degree of degenerative change for lumbar and thoracic spine radiographs (in percent, ± 95% confidence interval)

	Lumbar (in %)		Thoracic (in %)	
	Sensitivity	Specificity	Sensitivity	Specificity
No degenerative changes	50.0 (± 26.2)	99.3 (± 0.9)	50.0 (± 69.3)	100 (± 0.0)
Mild changes	67.3 (± 12.4)	98.5 (± 0.9)	42.3 (±19.0)	98.5 (± 1.3)
Moderate changes	65.9 (± 8.0)	96.2 (± 15.3)	61.6 (±11.1)	97.7 (± 1.5)
Severe changes	59.7 (± 8.8)	93.9 (± 1.9)	41.7 (±11.4)	97.8 (± 1.3)

## Discussion

Our study tested the performance of a commercially available AI software for fracture detection on spine radiographs. On a per vertebrae basis, the overall performance of the AI algorithm was mediocre, not measuring up to previously reported results on a per case basis, where often more than one fracture is present in the spinal radiograph, and the identification of a single of these fractures is enough for a true positive AI result [8, 9, 13]. In particular, thoracic spine radiographs showed a limited performance, concordant with the often difficult interpretation in real-life clinical practice due to overlay of the ribcage. Overall detection performance slightly improved when adding the ap-spine images for a second view, although grade of height loss cannot be accurately determined on these.

Slight compression fractures showed reduced sensitivities compared to more pronounced fractures, as expected. It should be noted that these fractures are also most likely to be missed by radiologists, the combination of AI and the radiologist may be a potential solution that could lead to overall improved fracture detection. Genant 2 and 3 fractures were identified at almost the same rate, likely there is no difference in performance after a certain threshold of vertebral height loss is reached. AI performance on wedge fractures was non-significantly better. Foreign material in the vertebra had a marked impact on performance, reducing both sensitivity and specificity.

Degenerative changes of the spine also had a marked influence on the AI’s performance. The very poor sensitivity for fractures in patients with no degenerative changes should be viewed with caution, as this dataset was very small, there is a large possible margin of error. Interestingly, mild changes in the thoracic spine performed as poorly as severe changes, with moderate changes showing a much better overall result, potentially this may be due to a bias in the training data originally used for the algorithm.

**Image 4:**
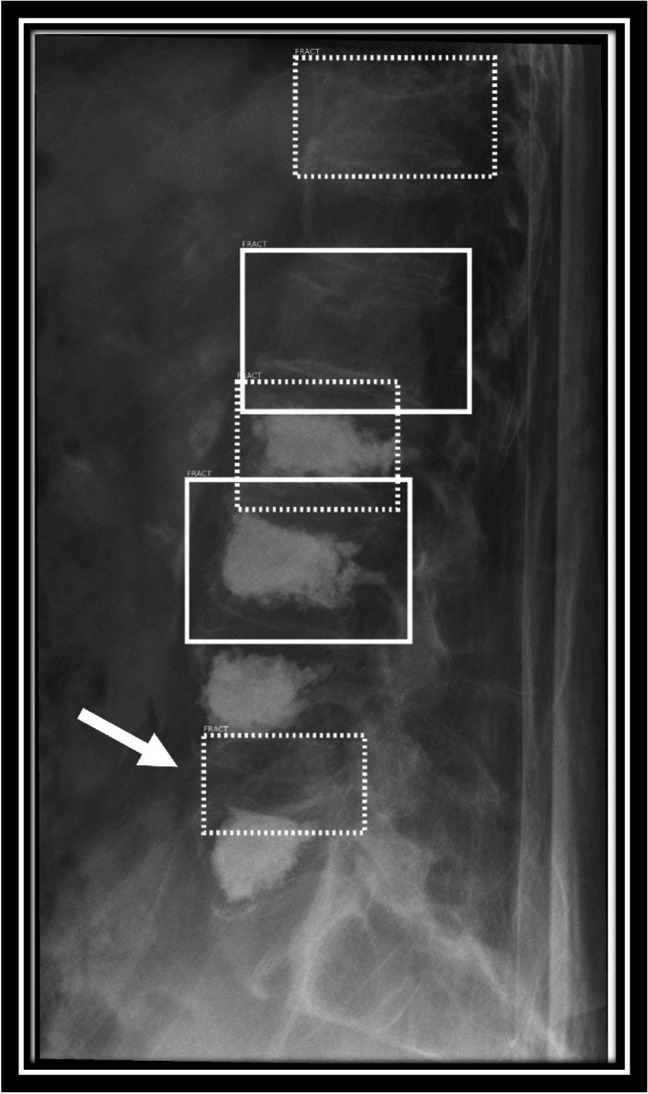
Lateral radiographs of the lumbar spine with markings by the AI software. The arrow indicates a dashed-line bounding box that the AI placed in the intervertebral space L4/5 as a region where a fracture is suspected, a false-positive result in this case. Such markings by the AI were disregarded for further analysis

**Image 5:**
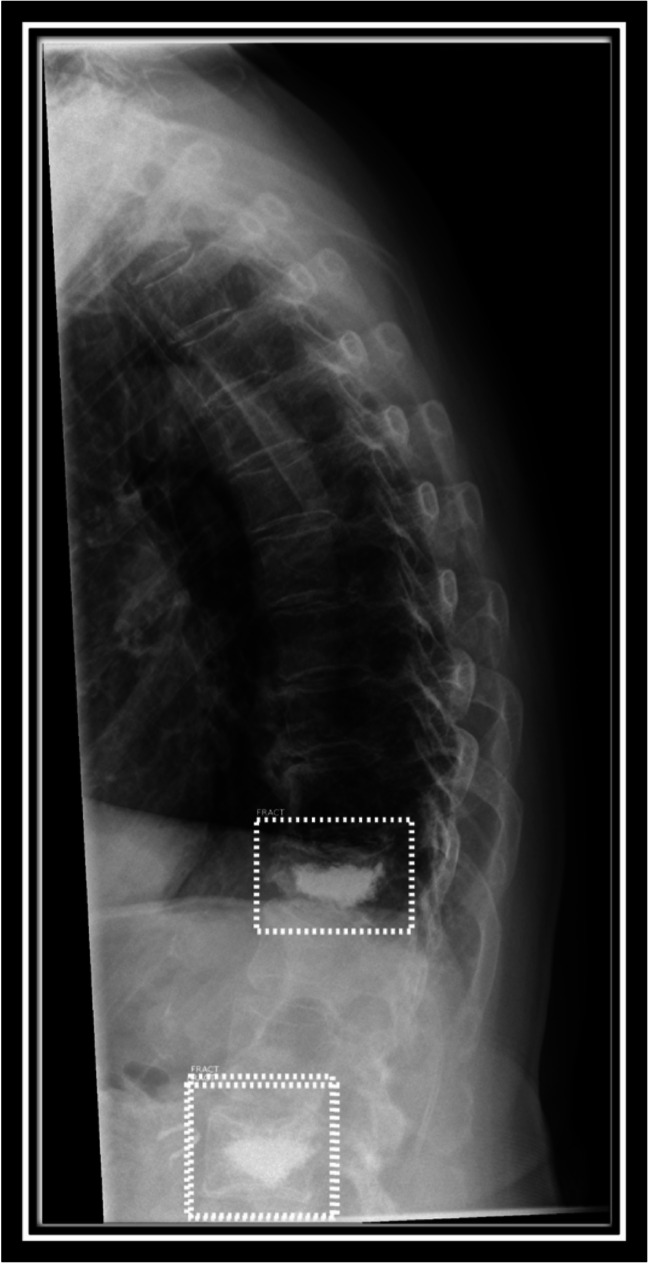
Lateral radiographs of the lumbar spine with markings by the AI software. Two dashed-line bounding boxes are erroneously placed around the L2 vertebrae as a potential fracture, where kyphoplasty was previously performed. All bounding boxes represent a true-positive, older fracture

**Image 6:**
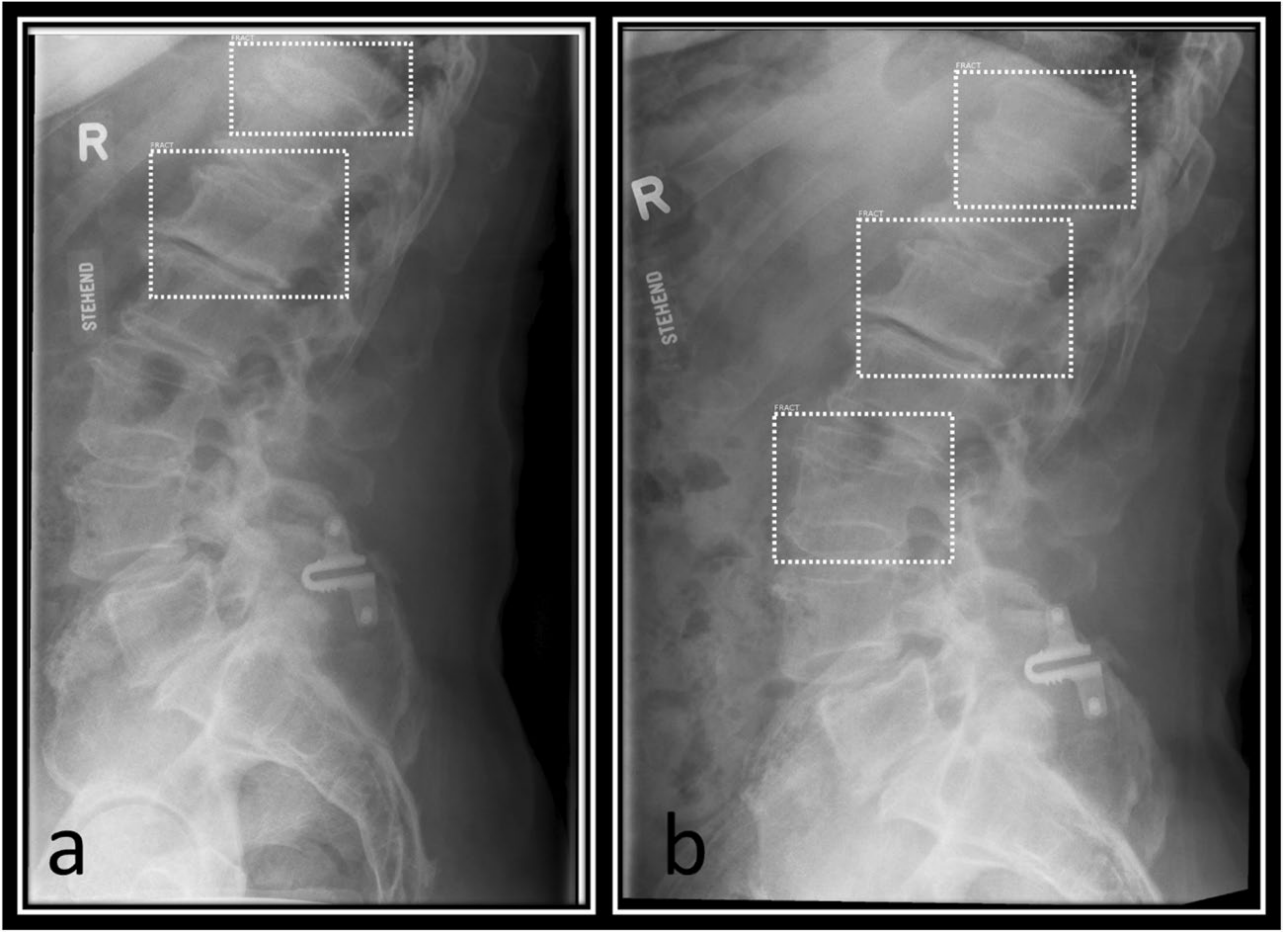
**a**, **b** from left to right: lateral spine radiographs of the lumbar spine of an identical patient in ante- and retroflexion with markings by the AI software showing a diversion in results. The AI marks an additional dashed-line bounding box on the L3 vertebra in one image, while only marking L1 and T11 in the other image of the same patient during the same exam, where additional functional imaging was obtained. The fractures in T11 and L1 showed true positive, grade 1 (L1) and grade 2 (T11) compression fractures; L3 shows a false positive

In some cases, the AI acted unexpectedly to the images sent for analysis. The AI rejected a small number of images completely, sometimes classifying thoracic spine radiographs as chest radiographs and lumbar spine radiographs as abdominal radiographs. This was most often the case in obese patients where the field of view for the radiograph was very wide, thereby including lots of surrounding tissue. These types of radiographs are not supported by the AI for fracture analysis. Other interesting “glitches” seen in the AI in a small number of cases were the marking of intervertebral space as a fracture (see Image 4), the marking of the same vertebra by two bounding boxes (see Image 5), and the marking of different vertebra as fractures in a case where additional functional imaging was available (see Image 6 a, b).

The reasons for the reduced performance of AI in cases where foreign material was included or in patients with severe degenerative change cannot be explicitly determined, as we only have limited insights into the development of the algorithm. In an external validation funded by Gleamer by Guermazi et al., some statistics for the software development are divulged. The company behind the AI notes that over 60,000 images were used in the training and validation from 22 different institutions. The training was augmented by random changes to the images, such as rotation and resizing. The AI is validated for fracture detection in “diagnostic quality” images; however, an explicit definition of this is not provided [8]. A further breakdown of the number of images per anatomical region or additional edge-cases such as with prior instrumentation are not provided. An increase in specific training data to fine-tune the model may mitigate the performance issues [14].

Other studies have tested different AI algorithms for vertebral fracture detection. Murata et al. were able to achieve a sensitivity of 84.7% and specificity of 87.3% for a proprietary detection algorithm used for research purposes. However, only patients with a single or no fracture were included and grade 1 compression fractures were excluded from the study [15]. Shen et al. were able to achieve sensitivities of about 84% with a very high specificity of up to 97% with a proprietary detection algorithm for vertebral fractures. Both algorithms were for research purposes only and did not have clearance by the proper authorities for clinical use as the algorithm tested in this study does. There was little difference in results between thoracic and lumbar spine fractures and mild fractures were detected at a rate of 73% in the external validation set [16].

There are some limitations to our study. Analysis of Genant classification were made by one radiologist only, a second opinion may improve accuracy. The presence or absence of a fracture was defined by the final report, it is possible that some fractures were missed or identified as false positives. Some subclassifications of the dataset include very small groups, leading to a degree of statistical uncertainness in these results. Cross-sectional imaging was available only for a partial set of the included studies, with which more accurate diagnoses and classifications could have been made. The addition of a radiologist’s impression with the AI software may improve overall detection rates and merits further research.

## Conclusions

Our study researched the performance of a commercially available AI algorithm for fracture detection on thoracic and lumbar spine radiographs on a large dataset with multiple parameters. On a per vertebrae basis, results were mediocre, in particular for subtle compression fractures. Overall sensitivity was 63.2% for lumbar spine fractures and 50.6% for thoracic spine fractures. For grade 1, fractures performance dropped to 52.5% in the lumbar spine and 42.4% in the thoracic spine. Factors such as prior surgery with orthopedic material in the bone and advanced degenerative changes of the spine further mitigate the AI’s performance, reaching levels below 60% sensitivity in the lumbar and 50% sensitivity in the thoracic spine.

## Data Availability

Data can be made available in an anonymized fashion upon reasonable request to the corresponding author.
